# Short‐term outcomes in patients with lung cancer‐associated acute ischemic stroke

**DOI:** 10.1111/1759-7714.14611

**Published:** 2022-08-18

**Authors:** Ruixia Wang, Peijun Xu, Jun Zhou, Yuanyuan Meng, Kun Men, Jinyuan Zhang, Wei Lu, Juanjuan Xue, Xin Li

**Affiliations:** ^1^ Department of Neurology The Second Hospital of Tianjin Medical University Tianjin China; ^2^ Tianjin Medical University Cancer Institute and Hospital National Clinical Research Center for Cancer, Key Laboratory of Cancer Prevention and Therapy, Tianjin's Clinical Research Center for Cancer Tianjin China; ^3^ Department of Neurology Qilu Hospital of Shandong University Dezhou Hospital Dezhou China; ^4^ Department of Neurology Shengli Oilfield Central Hospital Dongying China; ^5^ Department of Clinical Laboratory The Second Hospital of Tianjin Medical University Tianjin China; ^6^ Department of Network Information Center The Second Hospital of Tianjin Medical University Tianjin China

**Keywords:** acute ischemic stroke, lung cancer, outcomes, propensity score matching

## Abstract

**Background:**

To investigate the independent risk factors of poor short‐term outcomes in patients with lung cancer‐associated acute ischemic stroke (LCAIS) and use them to develop an index of prognosis LCAIS (pLCAIS) which could help clinicians identify patients at high risk for poor short‐term outcomes.

**Methods:**

We retrospectively enrolled patients with lung cancer‐associated acute ischemic stroke and employed the 90D modified Rankin cale (mRS) to divide them into two groups: good outcomes (score 0–2) and poor outcomes (score 3–6). Propensity score matching (PSM) was used to remove confounding factors, and multivariable logistic regression analysis was used to analyze the independent risk factors of pLCAIS. The receiver operating characteristic (ROC) and area under the ROC curve (AUC) developed a multiple model combining the independent risk factors of pLCAIS.

**Results:**

A total of 172 patients were included: 67 (38.9%) with good outcomes and 105 (61.1%) with poor outcomes. After using PSM, there were 33 cases in each group. The results showed that patients with poor short‐term outcomes were significantly higher in D‐dimer (OR = 1.001, 95% CI: 1.000–1.002, *p* = 0.048), CRP (OR = 1.078, 95% CI: 1.008–1.153, *p* = 0.028), and neutrophil count (OR = 14.673, 95% CI: 1.802–19.500, *p* = 0.012). The ROC curve, used to assess the diagnostic ability of binary classifiers, showed that the product of these three independent risk factors showed high sensitivity and specificity.

**Conclusion:**

In this study, we have identified three independent risk factors associated with poor short‐term outcomes in pLCAIS: higher NC, CRP, and D‐dimer levels. These findings may be helpful for clinicians in identifying poor short‐term outcomes patients.

## Graphical abstract

To investigate independent risk factors of poor short‐term outcomes in patients with lung cancer‐associated acute ischemic stroke (LCAIS) and use them to develop an index of prognosis LCAIS (pLCAIS) which could help clinicians identify patients at high risk for poor short‐term outcomes. A total of 172 patients with lung cancer‐ associated acute ischemic stroke from 4 centers were retrospectively included and divided into two groups using the 90D modified Rankin scale (mRS): good outcomes (mRS score 0–2) and poor outcomes (mRS score 3–6). The two groups of data were matched with a 1:1 propensity score, the caliper value was set to 0.02, and a total of 33 cases were matched in each group. To identify independent risk factors, all baseline variables with a *p* < 0.05 comparison between the two groups were included in a multivariate logistic regression analysis. Receiver operating characteristic (ROC) and area under the ROC curve (AUC) developed a multiple model incorporating independent risk factors for pLCAIS.

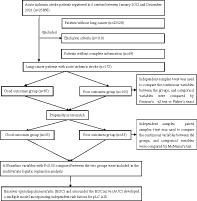

## INTRODUCTION

Cancer and ischemic stroke are two major diseases that pose a significant threat to human health. Stroke and lung cancer are two of the five leading causes of disability adjusted life years (DALYs) associated with an aging population in China.^1^ Patients with ischemic stroke ‐ the second‐leading cause of death in the world and the leading cause of adult disability in China ‐ often suffer life‐altering impacts to their physical, mental, and social capabilities. Stroke is also the most common complication of cancer,^2^ which can significantly aggravate the patient's condition and affect the prognosis.^3^ With modern treatments prolonging the 5‐year survival rates in China from 30.9%^4^ in 2003 to 40.5%^5^ in 2012, the incidence of thromboembolism is significantly higher than before. Many types of tumors have been shown to be associated with ischemic or hemorrhagic stroke.^6^ One study found that 6 months after tumor diagnosis, the risk of hemorrhagic stroke was 2.2%, and remained elevated even after 10+ years.^7^ Compared with hemorrhagic stroke, cancer survivors have a higher risk of ischemic stroke,^8^ cancer increases the risk of stroke at 3 years after the diagnosis of cancer, and the effect is maintained for 7 years.^9^

Lung cancer is a known high‐risk factor for cancer‐associated ischemic stroke.^10^ Cumulative incidence rates of stroke have previously been reported to be 5.1% in patients with lung cancer.^11^ Previous studies showed that elevated D‐dimer, fibrinogen, and CRP could be potential biomarkers of cancer‐associated acute ischemic stroke.^12^, ^13^

At present, studies on patients with lung cancer‐associated acute ischemic stroke mainly focus on the pathogenesis, clinical manifestations, imaging characteristics, etc., and there have been few studies on the prognosis of patients. Based on this, our study analyzed the risk factors of poor short‐term outcomes in patients with lung cancer who experienced acute cerebral infarction, which we hope will help clinicians identify such patients and improve their outcomes.

## METHODS

### Patient selection

**FIGURE 1:**
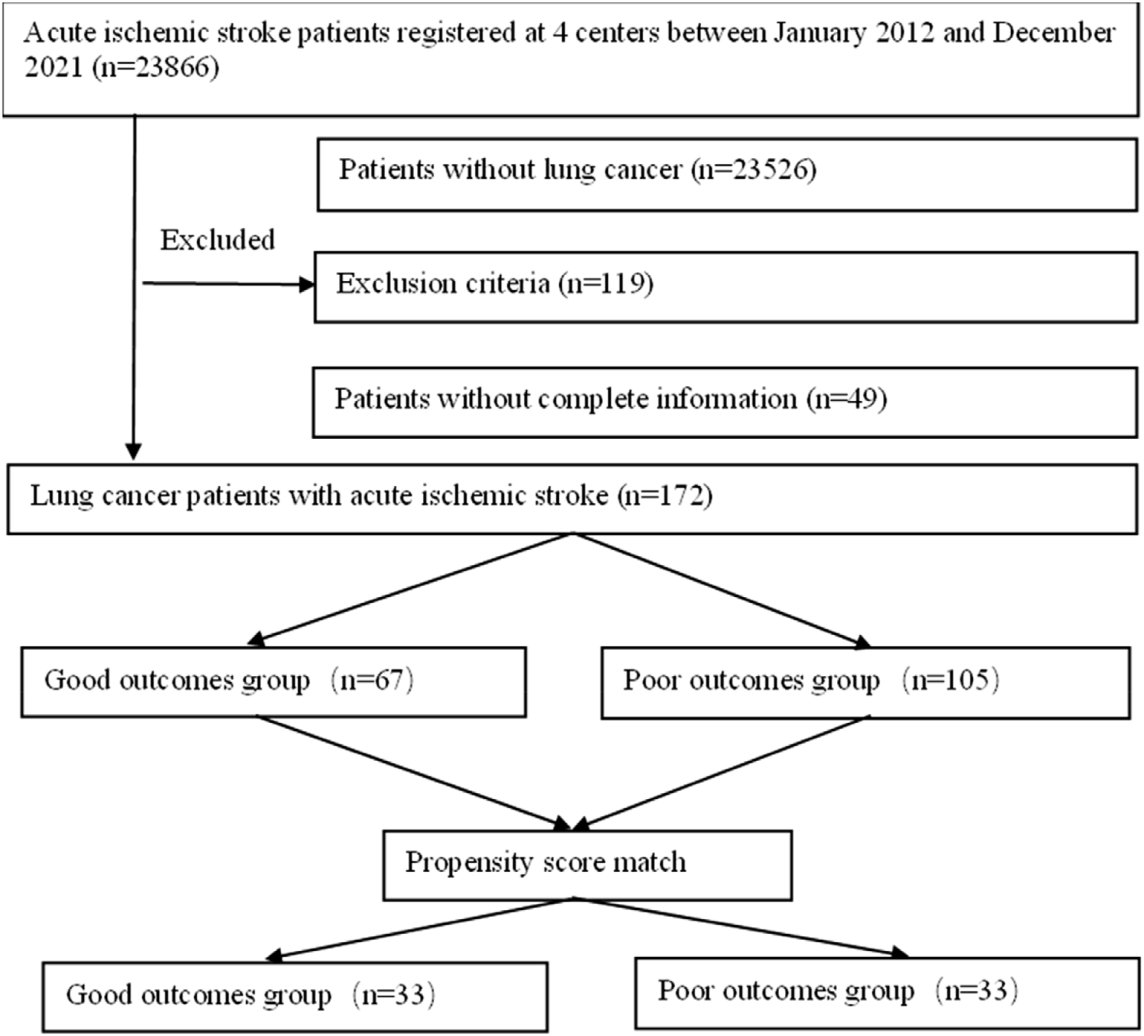
The flow chart of patients included in the current analysis

Data from patients with lung cancer‐associated acute ischemic stroke who were hospitalized in the Second Hospital of Tianjin Medical University, Tianjin Medical University Cancer Institute and Hospital, Qilu Hospital of Shandong University Dezhou Hospital and Shengli Oilfield Central Hospital from January 2012 to December 2021 were retrospectively collected. Inclusion criteria: (1) age > 18 years; (2) acute ischemic stroke patients met the diagnostic criteria of “China Guidelines for Diagnosis and Treatment of Acute Ischemic Stroke 2018,” confirmed by magnetic resonance imaging (MRI); (3) acute ischemic stroke occurred 3 months before diagnosis of non‐small cell lung cancer to 5 years after diagnosis; and (4) non‐small cell lung cancer patients had a complete history and pathological diagnosis. Exclusion criteria: (1) patients with intracranial cancers, including primary intracranial tumors or metastases; and (2) lung cancer patients who were not expected to survive for 3 months. A total of 172 patients were included: 85 males (49.4%) and 87 females (50.6%) with a mean age of 73.9 years. After propensity score matching, 66 patients were included in the study, with 33 patients in each group (Figure 1).

This study was conducted in accordance with the Declaration of Helsinki and approved by Ethical Review Committee. Approval Number: (KY2020K073).

### Collection of clinical data

All data were collected from paper medical records and the hospital information system, including lung cancer staging, treatment plan, comorbidities (diabetes, hypertension, hyperlipidemia, atrial fibrillation, history of smoking and drinking, history of stroke), laboratory markers: white blood cell count (WBC), neutrophil count (NC), red blood cell count (RBC), hemoglobin (HGB), platelets (PLT), D‐dimer (D‐D), fibrinogen (FIB), homocysteine (HCY), C‐reactive protein (CRP), carcinoembryonic antigen (CEA), carbohydrate antigen125 (CA125), cytokeratin fragment antigen 21–1(Cyfra21‐1), neuron‐specific enolase (NSE), whether antiplatelet therapy was received, whether anticoagulation therapy was received, and the location and number of cerebral infarction lesions in the diffusion‐weighted (DW) brain MRI images. The data related to acute ischemic stroke included the severity of neurological deficit using the National Institute of Health stroke scale (NIHSS) score and the etiological classification of cerebral infarction using Trial of Org10172 in Acute Stroke Treatment (TOAST) criteria. Follow‐up: after 90 days of discharge from the hospital, a neurologist conducted a face‐to‐face or telephone follow‐up in the cerebrovascular disease clinic and evaluated the patient's mRS score. The modified Rankin Scale (mRS) was used to evaluate the prognosis at 90 days after cerebral infarction. An mRS score of 0–2 was defined as a good outcome while an mRS score of 3–6 was defined as a poor outcome.^14^

### Statistical analysis

All statistical analyses were performed using SPSS version 25.0. The independent samples *t* test was used to compare the continuous variables between the groups, and categorical variables were compared by Pearson's *χ* ^2^ test or Fisher's exact test. The PSM method was used to reduce the effect of confounding factors and to make the comparison between two groups more reasonable. The two groups of data were matched with a 1:1 propensity score, the caliper value was set to 0.02, and a total of 33 cases were matched in each group. To determine the independent risk factors, all baseline variables with *p* < 0.05 compared between the two groups were included in the multivariate logistic regression analysis. The cutoffs with sensitivities and specificities were identified by ROC analysis. All *p*‐values were two‐sided and *p* < 0.05 was considered statistically significant.

## RESULTS

### Characteristics in patients with lung cancer

The time interval between lung cancer diagnosis and incidence of acute ischemic stroke in these 172 patients ranged from ranged from 15 days to more than 5 years. Patients had lung cancer ranging across all stages: stage I, 23; stage II, 14; stage III, 24; stage IV, 61; undetermined stage, 49. Among them, 29 patients received surgery, 17 received radiotherapy, 16 received chemotherapy, 61 received combined therapy, and 49 received no tumor therapy. At the same time, lung cancer markers: carcinoembryonic antigen (CEA), carbohydrate antigen 125 (CA125), cytokeratin fragment 19 (Cyfra21‐1), neuron‐specific enolase (NSE) and squamous cell carcinoma (SCC) were measured. There were statistically significant differences in stages IV, chemotherapy and NSE (*p* < 0.05), but after PSM, there were no significant differences in stage, treatment and markers (Table 1).

**TABLE 1:** Clinical characteristics in patients with lung cancer

Characteristics	Entire cohort			Propensity‐score matched cohort		
	Good outcomes group (*n* = 67)	Poor outcomes group (*n* = 105)	*p*‐value	Good outcomes group (*n* = 33)	Poor outcomes group (*n* = 33)	*p*‐value
**Cancer stages**						
Stage I	11 (16.4)	13 (12.4)	0.456	3 (9.1)	2 (6.05)	1.000
Stage II	7 (10.4)	7 (6.7)	0.377	1 (3.0)	2 (6.05)	1.000
Stage III	13 (19.4)	11 (10.5)	0.099	3 (9.1)	3 (9.1)	1.000
Stage IV	16 (23.9)	45 (42.8)	0.011*	11 (33.3)	15 (45.5)	0.481
Unstaged	20 (29.9)	29 (27.6)	0.752	15 (45.5)	11 (33.3)	0.481
**Treatments**						
Surgical treatment	9 (13.4)	20 (19.0)	0.338	4 (12.1)	3 (9.1)	1.000
Radiation Therapy	10 (14.9)	7 (6.7)	0.043	1 (3.0)	1 (3.0)	1.000
Chemotherapy	10 (14.9)	6 (5.7)	0.021*	3 (9.1)	3 (9.1)	1.000
Combination therapy	18 (26.9)	43 (41.0)	0.060	14 (42.5)	11 (33.3)	0.629
Untreated	20 (29.9)	29 (27.6)	0.742	11 (33.3)	15 (45.5)	0.481
**Markers**						
CEA	4.6 ± 1.6	5.1 ± 1.6	0.097	4.9 ± 2.0	5.1 ± 1.1	0.628
CA125	30.3 ± 10.2	31.6 ± 15.8	0.549	29.5 ± 9.7	34.2 ± 17.4	0.165
Cyfra21‐1	3.1 ± 1.1	3.2 ± 1.6	0.829	3.0 ± 1.2	3.0 ± 1.3	0.960
NSE	12.7 ± 5.4	16.8 ± 8.9	0.001*	13.1 ± 5.0	17.0 ± 8.8	0.052
SCC	1.6 ± 0.9	1.8 ± 1.0	0.132	1.8 ± 1.0	1.9 ± 1.0	0.702

* *p* < 0.05 indicates a statistically significant difference. Abbreviations: CA125, carbohydrate antigen 125; CEA, carcinoembryonic antigen; Cyfra21‐1, cytokeratin fragment antigen 21‐1; NSE, neuron‐specific enolase; SCC, squamous cell carcinoma.

### Univariate analysis of clinical characteristics in patients

Before PSM, among the 172 patients, 62 (36.0%) had hypertension and 35 (20.3%) had diabetes. No mural thrombus and no valve structural problems were found in all cases by echocardiography. Before PSM, statistically significant differences (*p* < 0.05) were found between the good and poor outcome groups for age, hypertension, dyslipidemia, smoking, history of stroke, RBC, WBC, NC, FIB, D‐dimer, HCY, CRP, baseline NIHSS score, In‐hospital mortality, 90D mortality and ≥3 arterial ischemic territories. In acute ischemic stroke treatment, TOAST classification, number of acute cerebral infarction lesions in one or two arterial ischemic territories were not significant. After PSM, significant differences (*p* < 0.05) were only found in WBC, NC, FIB D‐dimer, CRP, baseline NIHSS score, 90D mortality and ≥3 arterial ischemic territories (Table 2).

**TABLE 2:** The differences in characteristics between two groups before and after propensity score matching

Patient characteristics	Entire cohort, n (%)			Propensity‐score matched cohort, n (%)		
	Good outcomes group (*n* = 67)	Poor outcomes group (*n* = 105)	*p*‐value	Good outcomes group (*n* = 33)	Poor outcomes group (*n* = 33)	*p*‐value
Age (year)	71.7 ± 7.9	74.8 ± 7.8	0.012*	71.8 ± 8.1	73.6 ± 7.8	0.419
Male sex, n (%)	34 (50.7)	50 (47.6)	0.834	26 (56.5)	21 (45.7)	0.383
Hypertension, n (%)	31 (46.3)	31 (29.5)	0.001*	25 (54.3)	22 (47.8)	1.000
Diabetes, n (%)	12 (17.9)	23 (21.9)	0.526	12 (26.1)	12 (26.1)	1.000
CAD, n (%)	6 (9.0)	6 (5.7)	0.416	5 (10.9)	4 (8.7)	1.000
Dyslipidemia, n (%)	3 (4.5)	15 (14.3)	0.040*	3 (6.5)	4 (8.7)	1.000
Atrial fibrillation, n (%)	3 (4.5)	9 (8.6)	0.304	4 (8.7)	3 (6.5)	1.000
Smoking, n (%)	4 (6.0)	18 (17.1)	0.032*	3 (6)	8 (16)	1.000
Drinking, n (%)	4 (6.0)	7 (6.7)	0.856	4 (8)	5 (10)	0.625
History of stroke, n (%)	2 (3.0)	15 (14.3)	0.015*	4 (8)	6 (12)	1.000
RBC (×10^12^/l)	4.2 ± 0.8	3.6 ± 0.6	<0.001*	4.0 ± 0.8	3.6 ± 0.6	0.066
HGB (g/l)	117 ± 11.0	118 ± 10.8	0.641	117.9 ± 9.1	114 ± 9.6	0.155
WBC (10^9^/l)	5.6 ± 1.3	8.1 ± 2.2	<0.001*	5.7 ± 1.4	8.5 ± 2.5	<0.001*
NC (10^9^/l)	2.8 ± 0.8	5.2 ± 1.5	<0.001*	2.8 ± 0.8	5.3 ± 1.5	<0.001*
PLT (×10^9^/l)	248.6 ± 95.5	251.8 ± 95.6	0.981	224 ± 86.4	266 ± 106.7	0.067
FIB (g/l)	3.4 ± 0.8	3.9 ± 0.9	<0.001*	3.2 ± 0.8	3.9 ± 0.9	0.030*
D‐dimer (μg/l)^a^	548 (300, 860)	800 (500, 1565)	0.002*	600 (300, 900)	1130 (590, 3000)	0.006*
HCY (μ mol/l)	14.1 ± 5.6	17.9 ± 7.9	0.001*	14.8 ± 5.1	17.8 ± 8.1	0.053
CRP (μg/l)^a^	6 (4.0, 9.0)	12.0 (6.8, 21.0)	0.028*	5.7 (3.77， 11.0)	12.8 (7.8, 30)	0.022*
Baseline NIHSS score	3.9 ± 2.8	9.9 ± 5.1	<0.001*	3.6 ± 1.3	6.9 ± 3.2	0.001*
In‐hospital mortality	0 (0)	7 (6.6)	0.031*	0 (0)	0 (0)	–
90D mortality	5 (7.4)	26 (25.8)	0.001*	2 (6.1)	5 (15.2)	0.046*
**Treatments**						
Anticoagulant therapy	4 (6.0)	9 (8.6)	0.529	6 (13)	5 (10.9)	0.375
Antiplatelet therapy	47 (70.1)	85 (81.0)	0.102	37 (80.4)	36 (78.3)	0.302
Plasminogen therapy	6 (9.0)	5 (4.8)	0.273	1 (2.2)	2 (4.3)	0.620
Thrombolytic therapy	1 (1.5)	0 (0)	0.209	0 (0)	0 (0)	–
**TOAST types**						
Large atherosclerotic type, n (%)	21 (31.3)	26 (24.8)	0.345	14 (30.4)	12 (26.1)	1.000
Small artery occlusion type, n (%)	7 (10.4)	7 (6.7)	0.377	4 (8.7)	5 (10.9)	0.625
Cardiogenic, n (%)	3 (4.5)	9 (8.6)	0.304	4 (8.7)	3 (6.5)	1.000
Other cause type, n (%)	3 (4.5)	2 (1.9)	0.327	3 (6.5)	3 (6.5)	1.000
Unexplained type, n (%)	33 (49.3)	62 (59.0)	0.208	21 (45.7)	24 (48.9)	0.454
**Ischemic territory pattern (DWI)**						
One arterial territory, n (%)	17 (25.4)	17 (16.3)	0.149	7 (15.2)	11 (23.9)	0.508
Two arterial territories, n (%)	22 (32.8)	20 (19.0)	0.073	16 (34.8)	10 (21.7)	0.210
≥3 arterial territories, n (%)	28 (41.8)	67 (63.8)	0.005*	23 (50)	24 (52.2)	0.031*

* *p* < 0.05 indicates a statistically significant difference.

^a^Medians (interquartile).

Abbreviations: CAD, coronary artery disease; CRP, C‐reactive protein; DWI, diffusion‐weighted imaging; FIB, fibrinogen; HGB, hemoglobin; HCY, homocysteine; NC, neutrophil count; PLT, platelets; RBC, red blood cell count; WBC, white blood cell count.

### Multivariate logistic regression analysis of risk factors affecting poor short‐term prognosis in patients with lung cancer‐associated acute ischemic stroke after PSM

Multivariate logistic regression analysis showed that increased D‐dimer (OR = 1.001, 95% CI: 1.000–1.002, *p* = 0.048), CRP (OR = 1.078, 95% CI: 1.008–1.153, *p* = 0.028), and neutrophil count (OR = 14.673, 95% CI: 1.802–19.500, *p* = 0.012) were the significant three risk factors for poor short‐term outcomes in patients with lung cancer‐associated acute ischemic stroke (Table 3).

**TABLE 3:** Predictors of prognostic patients with lung cancer‐associated acute ischemic stroke by multivariable logistic regression analyses after PSM

Factors	β	SE	Wals	*p*‐value	OR value	95% CI
WBC	0.019	0.855	0.001	0.982	0.981	0.184–5.237
NC	2.686	1.070	6.301	0.012*	14.673	1.802–19.500
FIB	0.819	0.572	2.046	0.153	2.268	0.739–6.964
D‐dimer	0.001	0.001	3.911	0.048*	1.001	1.000‐1.002
CRP	0.075	0.034	4.849	0.028*	1.078	1.008‐1.153
≥3 arterial territories	0.233	1.145	0.041	0.839	1.262	0.134–11.908
Constants	−15.240	5.038	9.150	0.002*	0.000	–

* *p* < 0.05 indicates a statistically significant difference.

Abbreviations: CRP, C‐reactive protein; FIB, fibrinogen; NC, neutrophil count; WBC, white blood cell count.

### ROC curve

The ROC curve showed that the cutoff value of D‐dimer was 2088 mg/l, the AUC value was 0.789 (95% CI: 0.680–0.898), the sensitivity was 54.5%, and the specificity was 97.0%; the cutoff value of CRP was 7.55 mg/l, AUC value was 0.839 (95% CI: 0.737–0.940), sensitivity was 87.9%, specificity was 75.8%; the cutoff value of NC was 3.25*10^9^/l, AUC value was 0.888 (95% CI: 0.810–0.966), with a sensitivity of 90.9% and a specificity of 69.7%.

**FIGURE 2:**
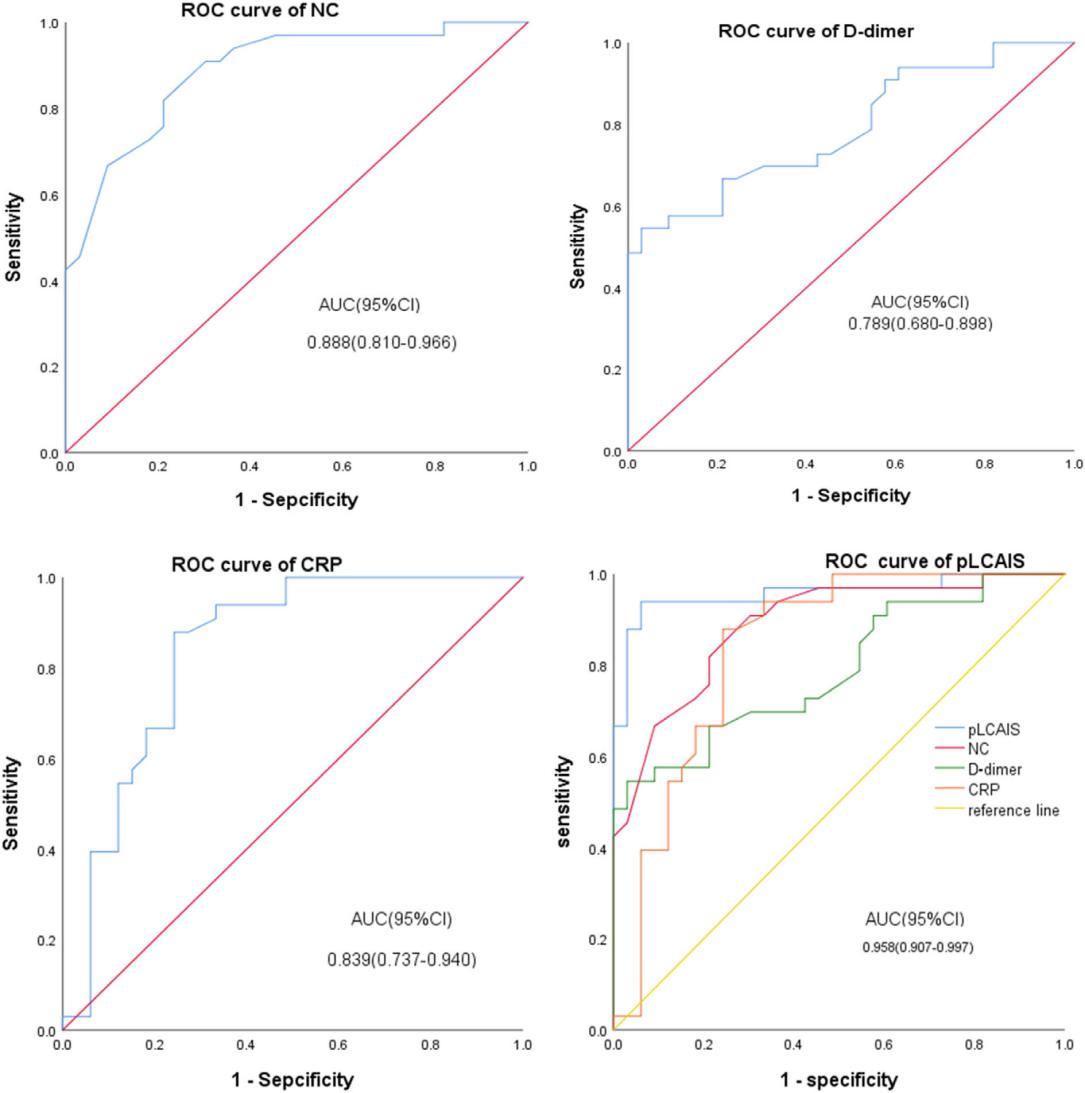
Receiver operating characteristic (ROC) curve for D‐dimer, CRP, NC and multiple models of pLCAIS to predict prognosis of lung cancer‐associated acute ischemic stroke. Abbreviations: AUC, area under the curve; CI, confidence interval; ROC: receiver operating characteristic

The product of the three risk factors can be described by the following equation: logistic (*p*) = In(*p*/[1 − *p*]) = β0 + β1X1 + β2X2 + β3X3, where *p* is the probability of poor short‐term outcomes of lung cancer‐associated acute ischemic stroke, X1 is the D‐D level, X2 is the CRP level, and X3 is the neutrophil count. ROC regression analysis showed that the product of the area under the curve (AUC) of the three risk factors was the largest, with an AUC value of 0.958 (95% CI: 0.907–0.997), a sensitivity of 93.9%, and a specificity of 93.9%, which was called this study pLCAIS index (Table 4, Figure 2).

**TABLE 4:** Receiver operating characteristic curve analysis of prognostic factors

Factors	AUC	95% CI	Sensitivity	Specificity
D‐dimer	0.789	0.680–0.898	54.5%	97.0%
CRP	0.839	0.737–0.940	87.9%	75.8%
NC	0.888	0.810–0.966	90.9%	69.7%
pLCAIS	0.958	0.907–0.997	93.9%	93.9%

Abbreviations: AUC, area under the curve; CI, confidence interval; CRP, C‐reactive protein; NC, neutrophil count; pLCAIS, prognosis of lung cancer associated acute ischemic stroke.

## DISCUSSION

The data of this study were processed by PSM to remove confounding factors and showed that D‐dimer, CRP, neutrophil count, and baseline NIHSS score were associated with poor short‐term outcomes in patients with lung cancer‐associated acute ischemic stroke, and the difference in 90D mortality between the two groups was statistically significant. It is suggested that improving the hypercoagulable state and inflammatory response of patients can lead to better short‐term outcomes.

The outcomes of patients with cancer‐associated acute ischemic stroke are poor overall.^15^ Among the 172 patients included in this study, 105 patients (61.1%) had poor outcomes, seven (4.1%) patients died during hospitalization, and 31 (18.0%) patients died during 90D follow‐up, rates that are consistent with other reports.^16^ After propensity score matching was performed, a total of 33 pairs of patients were successfully matched for further analysis. Studies have shown that traditional cerebrovascular disease risk factors in patients with cancer‐associated acute ischemic stroke, such as hypertension, diabetes, hyperlipidemia and smoking are low. Before PSM, we also showed that age, hypertension, dyslipidemia, smoking and history of stroke had a statistically significant difference between the two groups, but after PSM, there was no statistical difference in traditional cerebrovascular disease risk factors between the two groups. Before PSM, univariate analysis showed that there were significant differences between the good outcome group and the poor outcome group in terms of tumor stage (for stage IV), chemotherapy, and NSE (*p* < 0.05). After PSM, the above differences were not statistically significant. This may indicate that tumor staging and treatment methods do not have much influence on the short‐term prognosis of patients with lung cancer‐associated acute ischemic stroke, which may be related to the mRS as the evaluation standard.

The main treatment methods for acute cerebral infarction are thrombolytic, anticoagulation and antiplatelet aggregation therapies. Previous studies have shown that direct oral anticoagulants significantly decrease the risk of cancer‐associated thrombosis recurrence,^17^and anticoagulation therapy might prevent the recurrence of cancer‐associated stroke^18^ and can improve its short‐term prognosis.^15^, ^19^ There have also been studies comparing antiplatelet and oral anticoagulant therapy to prevent recurrence in tumor patients with cerebral infarction; however, there was no significant difference between the two methods, but the aspirin group was found to have a lower risk of bleeding than with oral rivaroxaban anticoagulant therapy.^20^ However, our study showed no significant difference among the three treatments, although this may be related to the relatively few patients who received thrombolytic and anticoagulant therapy in our dataset.

Before PSM, the RBC, WBC, NC, FIB, D‐D, HCY, CRP, baseline NIHSS score, in‐hospital mortality, 90D mortality and ≥3 arterial ischemic territory were significantly different between the two groups (*p* < 0.05). After PSM, only the WBC, NC, D‐D, FIB, CRP, baseline NIHSS score, ≥3 arterial ischemic territory and 90D mortality were significantly different between the two groups (*p* < 0.05). Multivariate logistic regression analysis showed D‐dimer (OR = 1.001, 95% CI: 1.000–1.002, *p* = 0.048), CRP (OR = 1.078, 95% CI: 1.008–1.153, *p* = 0.028), and neutrophil count (OR = 14.673, 95% CI: 1.802–19.500, *p* = 0.012) were risk factors for poor short‐term outcomes in patients with lung cancer‐associated acute ischemic stroke.

Cancer cells can activate the coagulation system, and cancer patients often have elevated levels of D‐dimer, which is a risk factor for cancer‐associated cerebral infarction^21^ and a predictor of early neurological deterioration.^22^ CRP is a reactive protein in the acute of inflammation phase, which can promote atherosclerosis and thrombosis. It has a certain significance on the severity and prognosis of cerebral infarction. CRP levels have been shown to be elevated in patients with cancer and cerebral infarction compared with patients with cerebral infarction but without cancer. At the same time, a previous study highlighted that if patients with cerebral infarction are higher in age, have a history of smoking, and have elevated CRP, clinicians should be alert as to whether the patients have an underlying malignancy.^23^ Therefore, CRP has also been explored as a potential marker for the diagnosis of Trousseau syndrome in patients with cerebral infarction.^12^ Higher D‐dimer and CRP levels are associated with cancer, especially lung cancer,^24^ and previous studies have also shown that patients with lung cancer have a higher risk of stroke.^25^ It has also been shown that mucins secreted by cancer cells bind to platelets and *p*‐selectin, triggering the mutual activation of platelets and neutrophils and leading to a hypercoagulable state promoting thrombosis. At the same time, neutrophils release extracellular meshwork, called neutrophil extracellular traps (NETs),^26^ which are associated with hypercoagulable states and thrombotic diseases in cancer patients.^27^ Cancer can induce increased peripheral blood neutrophil counts in solid tumor models, and increased neutrophil counts may lead to increased release of NETs in blood vessels, which may further promote a hypercoagulable state by stimulating platelet activation. As such, neutrophil NETs are considered to be a novel procoagulant mechanism in cancer patients.^28^

This study found that the elevation of D‐dimer, CRP, and NC may all play a role in the outcomes of patients with lung cancer‐associated acute ischemic stroke. While the three independent risk factors may each play their respective roles in the development of pLCAIS, they may also play a coordinated role. Their elevation can also be seen in other diseases or pathological states, and it is difficult to predict the prognosis of patients from any of these factors alone. We developed a multiple model of pLCAIS by combining three risk factors to predict the outcomes of patients with lung cancer‐associated acute ischemic stroke. We found that the multiple model of pLCAIS had the largest AUC ROC compared to each of the three individual risk factors, with high sensitivity and specificity, indicating that the multiple model of pLCAIS could be a specific biomarker of pLCAIS and may serve as a predictor of the development of pLCAIS.

The role of the pLCAIS Index in pLCAIS patients will need to be confirmed in future studies with larger sample sizes and more biomarkers. Nevertheless, our study provided a meaningful method to further the investigation of the index of pLCAIS in future studies.

The present study had some limitations. First, there is a potential risk of selection bias owing to the retrospective design, and differences in patient characteristics between the two groups were not completely overcome even after PSM, which could influence prognosis. Second, the relatively small sample size. Given the limitations of retrospective data, the relevant findings should be confirmed through prospective clinical studies with a large sample size.

In conclusion, our findings suggest that the poor outcomes of pLCAIS may be induced by elevated D‐D, CRP and neutrophils. The pLCAIS index, which serves as a novel biomarker of pLCAIS, needs to be confirmed in future studies.

## AUTHOR CONTRIBUTIONS

Conceptualization: Ruixia Wang, Peijun Xu, Xin Li; Data collection: Ruixia Wang, Peijun Xu, Jun Zhou, yuanyuan Meng, Kum Men, Jinyuan Zhang; Data analysis: Ruixia Wang, Peijun Xu, Wei Lu, Juanjuan Xue; Writing‐original draft: Ruixia Wang; Writing‐review & editing: Xin Li.

## CONFLICT OF INTEREST

The authors declare there are no potential conflicts of interest.
